# Post-stroke seizures, epilepsy, and mortality in a prospective hospital-based study

**DOI:** 10.3389/fneur.2023.1273270

**Published:** 2023-12-01

**Authors:** Sofia Freiman, W. Allen Hauser, Flora Rider, Sofia Yaroslavskaya, Olga Sazina, Elena Vladimirova, Igor Kaimovsky, Alexander Shpak, Natalia Gulyaeva, Alla Guekht

**Affiliations:** ^1^Moscow Research and Clinical Center for Neuropsychiatry of the Healthcare Department of Moscow, Moscow, Russia; ^2^Laboratory of Functional Biochemistry of the Nervous System, Institute of Higher Nervous Activity and Neurophysiology, Russian Academy of Sciences, Moscow, Russia; ^3^Gertrude H. Sergievsky Center, College of Physicians and Surgeons, New York, NY, United States; ^4^Buyanov City Hospital of the Healthcare Department of Moscow, Moscow, Russia; ^5^Konchalovsky City Hospital of the Healthcare Department of Moscow, Moscow, Russia; ^6^The Fyodorov Eye Microsurgery Federal State Institution, Moscow, Russia; ^7^Pirogov Russian National Research Medical University, Moscow, Russia

**Keywords:** post-stroke epilepsy, seizures, stroke outcome, watershed infarction, ischemic stroke, post-stroke recovery, risk factors

## Abstract

**Background and objectives:**

Post-stroke epilepsy (PSE) is a significant concern in the elderly population, with stroke being a leading cause of epilepsy in this demographic. Several factors have shown consistent associations with the risk of developing PSE, including cortical lesions, initial stroke severity, younger age, and the occurrence of early seizures. The primary objectives of this study were two-fold: (1) to determine the incidence of PSE and (2) to identify the risk factors associated with PSE in a prospective cohort of post-stroke patients.

**Methods:**

A prospective single-hospital study was conducted, involving patients diagnosed with acute ischemic and hemorrhagic stroke. The patients were followed up for 2 years (or until death) from the time of admission. Data about seizure occurrence and recurrent stroke were collected. Kaplan–Meyer curves were used for the assessment of PSE incidence and mortality. Possible predictors of PSE and mortality were selected from between-group analysis and tested in multivariable regressions.

**Results:**

Our study enrolled a total of 424 patients diagnosed with acute stroke. Among them, 97 cases (23%) experienced early post-stroke seizures, and 28 patients (6.6%) developed PSE. The cumulative risks of developing PSE were found to be 15.4% after hemorrhagic stroke and 8.7% after ischemic stroke. In multivariable fine and gray regression with competitive risk of death, significant predictors for developing PSE in the ischemic cohort were watershed infarction (HR 6.01, 95% CI 2.29–15.77, *p* < 0.001) and low Barthel index at discharge (HR 0.98, CI 0.96–0.99, *p* = 0.04). Furthermore, patients who eventually developed PSE showed slower recovery and presented a worse neurologic status at the time of discharge. The in-hospital dynamics of the National Institutes of Health Stroke Scale (NIHSS) were significantly worse in the PSE group compared to the non-PSE group (*p* = 0.01).

**Discussion:**

A higher proportion of cases experienced early seizures compared to what has been commonly reported in similar studies. Watershed stroke and low Barthel index at discharge were both identified as independent risk factors of PSE in ischemic strokes, which sheds light on the underlying mechanisms that may predispose individuals to post-stroke epilepsy after experiencing an ischemic stroke.

## Highlights

- Independent risk factors for post-stroke epilepsy following ischemic strokes include the watershed mechanism and a low Barthel index at discharge.
- Slow post-stroke recovery may contribute to the development of post-stroke epilepsy.
- The frequency of early post-stroke seizures, as detected by trained staff, relatives, and ambulance teams, was found to be 23% among all stroke cases.

## Introduction

Stroke stands as a prominent precursor to newly diagnosed epilepsy in the elderly population. Post-stroke epilepsy (PSE) accounts for 30–50% of newly diagnosed epilepsy cases in individuals aged 60 years and above (1–3). The cumulative risk of developing PSE within 1–5 years following an ischemic stroke is estimated to be 2–5%, which can elevate to 12.5% with a prolonged follow-up period of 10–12 years (4–8). The incidence of epilepsy after a hemorrhagic stroke is comparatively higher, reaching 6–13% within a few years after the index event (9–11).

Notably, PSE substantially decreases the quality of life not only for the affected patient but also for their family members. Additionally, PSE has been associated with increased mortality rates following a stroke (12).

With various degrees of certainty, several risk factors are demonstrated to increase the risk of developing PSE. Among the well-defined risk factors associated with PSE are hemorrhagic stroke, cortical lesions, initial stroke severity, young age, and occurrence of seizures during the acute stroke period (13). However, other risk factors, such as a cardioembolic mechanism or involvement of specific circulation territories, remain debated (13). Furthermore, even for the well-established risk factors, contradictory data exists, which can be attributed to differences in study characteristics as well as the complexity of underlying biological mechanisms.

Despite the extensive body of experimental and clinical studies, the exact biological mechanism underlying the formation of epileptic foci after a stroke remains unclear. This may explain why effective strategies to prevent PSE have not yet been developed even though significant progress has been made in stroke treatment, leading to improved post-stroke surveillance and neurological recovery. To advance our understanding and develop effective preventive strategies for PSE, there is a crucial need for further clinical and experimental studies. These studies should aim to elucidate the roles of both well-defined and potential predictors in the development of PSE and unravel the intricate mechanisms contributing to this condition.

Based on our previous (unpublished) research, we assumed that a number of early post-stroke seizures (ES) may be overlooked through routine clinical practice. To address this, we carefully designed our methodology to ensure a meticulous identification of early seizures (ES) in our cohort. As a result, we observed an incidence of ES higher than reported by most of the published studies. Our data revealed a robust association between ES and in-hospital mortality, indicating its clinical significance. In this study, we identified independent risk factors of epilepsy after ischemic stroke and incidence of epilepsy after ischemic stroke and stroke with hemorrhagic component. Our research gave us a deeper understanding of the factors influencing the occurrence of post-stroke epilepsy.

## Methods

### Patients

Patients with acute stroke admitted to the stroke unit of Buyanov City Hospital (Moscow) from 1 January 2013 to 31 December 2016 were enrolled in this study. We excluded 19 patients who underwent thrombolysis in the acute stroke period since this approach to stroke treatment was not yet well established at our hospital during the study period.

To ensure a more focused investigation on post-stroke epilepsy (PSE) and post-stroke mortality, patients with pre-existing epilepsy were excluded as were people with comorbid catastrophic illnesses. The latter included patients in late stages of malignancy, severe myocardial infarction associated with stroke, and terminal stages of renal disease. By excluding these specific groups, we aimed to minimize the potential impact of other serious diseases on both mortality rates and the occurrence of epilepsy in our study cohort.

### Definitions, measures, and data collection

Stroke was defined according to the World Health Organization criteria as a rapidly developing neurological deficit lasting for more than 24 h and confirmed by CT/MRI. The Glasgow Coma Scale (GCS) and the National Institute of Health Stroke Scale (NIHSS) were evaluated in the acute period at days 1, 3, 7, and at discharge (on days 21–28 after stroke). The data about risk factors (obesity, dyslipidemia, diabetes, atrial fibrillation, hypertension, ischemic heart disease, and myocardial infarction), lifestyle habits (smoking and alcohol consumption), standard laboratory parameters, and medications were collected for all patients.

Seizures were defined according to the International League Against Epilepsy (ILAE) guidlines (14, 15). A seizure occurring within the first 7 days of the onset of stroke was categorized as an early seizure (ES), while a seizure occurring on day 8 or later was classified as a late seizure. Standardized clinical evaluation including Responsiveness in Epilepsy Scale (RES) (16) was performed by trained medical staff in all cases when a seizure occurred in the hospital. Status epilepticus was defined as seizure activity or a series of seizures lasting for more than 5 min (17). All late seizures that occurred within the first month after stroke were diligently examined to exclude provoked seizures. The occurrence of at least one late unprovoked seizure was considered as post-stroke epilepsy (18, 19), which is consistent with ILAE recommendation (18, 19).

The presence of cortical involvement was assessed by CT/MRI. Ischemic stroke cases were classified according to the vascular territory involved. Watershed stroke was identified when infarcts were located in borderzone areas at the junction between two main arterial territories (20, 21).

Regarding lifestyle habits, individuals who were current smokers or ex-smokers with a non-smoking period of < 6 months were categorized as “Smoker.” A “Heavy drinker” was defined as someone with alcohol consumption of more than 600 g of pure alcohol per month.

### Follow-up

Outcomes of the study, which included seizure occurrences and mortality, were gathered throughout the entire duration of hospitalization and over a 2-year follow-up period. This data collection was conducted by a neurologist at in-person follow-up visits or through structured telephone interviews.

### Seizure ascertainment

The information about seizures before admission was obtained from medical records, relatives, and patients themselves. Additionally, the records and communication of the ambulance team were carefully reviewed to gather any relevant data regarding seizures prior to hospitalization. A neurologist with expertise in epilepsy monitored each patient through the whole in-hospital stay: in the stroke unit and subsequently after transfer to the inpatient neurology unit. In cases of uncertainty about a reported seizure, a qualified epileptologist available 24/7 in the Buyanov City Hospital was called to verify the seizure. The staff was specially trained to record all seizure episodes not only seizures with generalized features (focal to bilateral tonic-clonic seizures or unknown onset) but also focal aware and focal with impaired awareness seizures (14, 15). To ensure consistency and reliability, all medical records were later reviewed by a neurologist and/or epileptologist.

### Follow-up and post-stroke epilepsy

During the follow-up period, scheduled visits were conducted at specific time points, including 3, 6, 12, 18, and 24 months after the initial stroke event. At each follow-up visit, a neurologist collected information about neurological status, seizures, medical treatment, recurrent stroke, and death through in-person visits or telephone interviews with patients themselves or their relatives. To assess the occurrence of seizures during the follow-up period, a structured questionnaire was utilized. The neurologist used this questionnaire to inquire specifically about any seizure events experienced by the patients during the period after a previous examination.

The primary endpoint of the study was the development of PSE, aiming to identify patients who experienced epileptic seizures during the follow-up period. The secondary endpoint was overall mortality, with the aim of determining the short-term and long-term survival outcomes after a stroke.

### Statistical analysis

Univariable and multivariable statistics were performed for gender, age, risk factors, stroke type, scales for neurological state and functional recovery, and available laboratory data. Univariable tests were performed for preliminary selection parameters for multivariable analysis in Fine and Gray and Cox regressions. For categorical variables, between-group analysis was conducted using the chi-square test with Yates's correction. For ranked values and laboratory data with abnormal distribution, the Mann–Whitney *U*-test was applied. Student's *t*-test was used for laboratory data that followed a normal distribution. All statistical tests were two-tailed. The normality of distribution was estimated with the Kolmogorov–Smirnov test.

Risk factors associated with PSE were identified using the Fine and Gray model considering the competitive risk of death. Furthermore, to determine the risk factors associated with short-term and long-term mortality, Cox proportional hazard models were employed. Hazard ratios (HR) and their corresponding 95% confidence intervals (CI) were calculated as part of the analysis.

The cumulative risks of PSE and mortality were estimated using Kaplan–Meier analysis. The first occurrence of a late-onset post-stroke seizure was considered the qualifying endpoint for PSE assessment, while death was the endpoint for mortality assessment. Patients who dropped out of the study for other reasons before reaching the endpoint were censored. To compare the incidence of PSE and overall survival between different cohorts, the log-rank test was utilized. To examine the time course of the National Institute of Health Stroke Scale (NIHSS) scores, ANOVA with mixed design was employed. This analysis enabled the comparison of NIHSS scores at different time points during the study period. For *post-hoc* comparisons, the false discovery rate (FDR) correction method, specifically the Benjamini/Hochberg method, was applied.

### Ethical approval

Ethical approval for this study was obtained from the Medical Ethical Committee of Buyanov City Hospital (approval number 14-2011). All participants were included in this study after an informed consent was signed by the patient or a responsible party.

### Data availability

Anonymized data not published in this article may be shared upon request of the qualified investigator.

## Results

**Figure 1:**
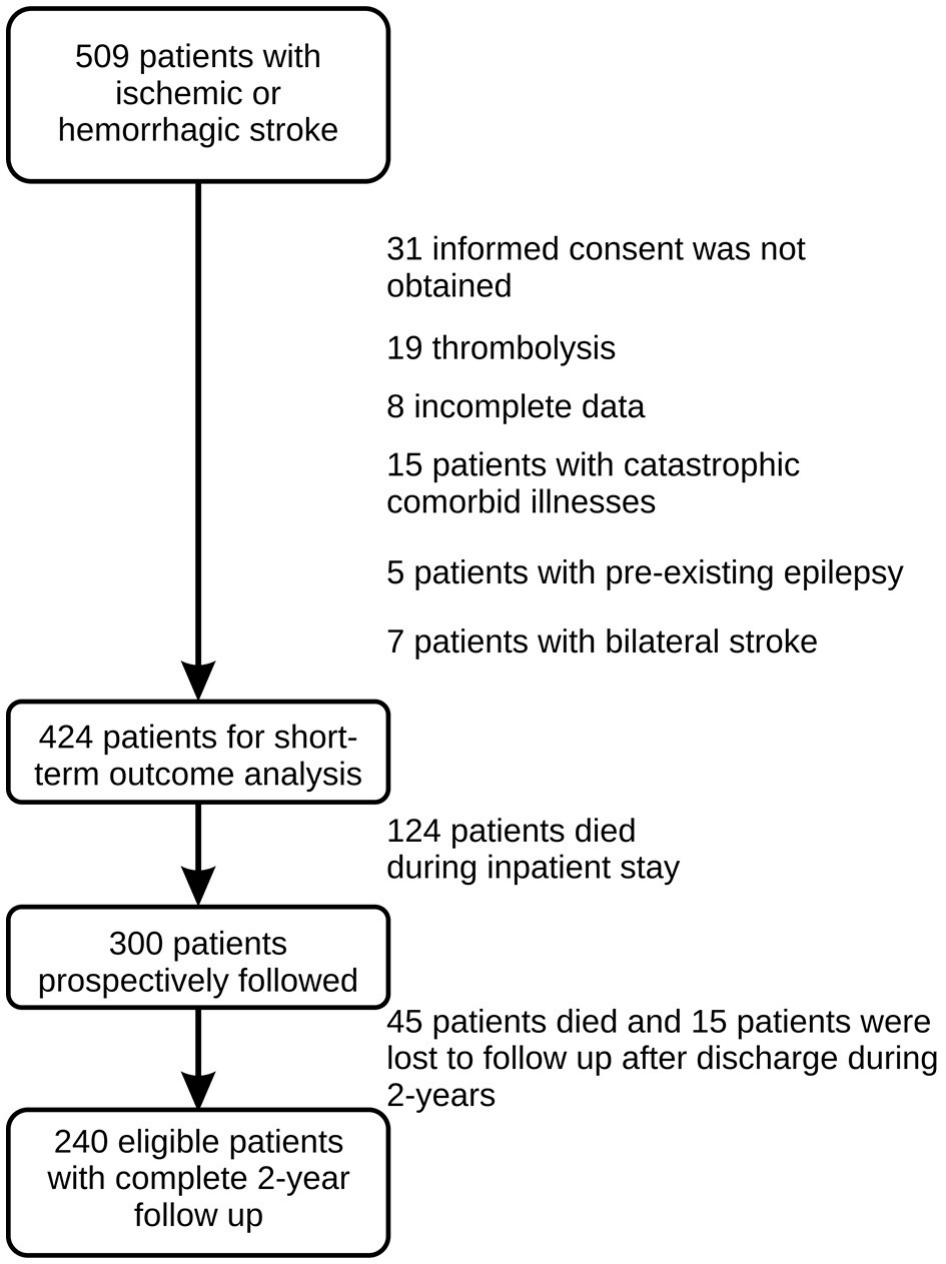
Flowchart for study design and number of patients.

Of the 509 patients who fulfilled eligibility for inclusion criteria for this study, 5 were later excluded because of a previous history of unprovoked seizures, and 80 patients were excluded for other reasons (Figure 1).

After the exclusions, the remaining cohort comprised 424 participants, whose characteristics are summarized in Table 1. Of the 424 enrolled patients, 223 (53%) were men, and 201 (47%) were women. The average age of the participants was 66.8 (±11.1) years. The distribution of diagnoses within the cohort was as follows: ischemic stroke in 343 patients (80.9%), ischemic stroke with hemorrhagic transformation in 16 patients (3.8%), intracranial parenchymal hemorrhage in 37 patients (8.7%), and subarachnoid hemorrhage in 28 patients (6.6%). Further details regarding the characteristics of the study participants can be found in Table 1.

**Table 1:** Characteristics of the stroke cohort (*n* = 424).

	**Post-stroke patients**, ***N*** = **424 (100%)**	
Age, mean (±SD)	66.8	±11.1
Male sex, *N* (%)	223	52.6%
Ischemic stroke	343	80.9%
Hemorrhagic stroke	81	19.1%
First-ever, *N* (%)	348	82.1%
NIHSS at admission, median (IQR^**^)	9.0	5.0–15.0
Early-onset seizure (0–7 days), *N* (%)	97	22.9%
Focal, *N* (%)	76	17.9%
Seizures with generalized features (focal to bilateral tonic-clonic seizures or unknown onset), *N* (%)	18	4.2%
Status epilepticus, *N* (%)	3	0.7%
**Vascular risk factors**, ***N*** **(%)**		
Diabetes mellitus	172	40.6%
Dyslipidemia	199	46.9%
Hypertension (160/100 and above)	381	89.9%
Atrial fibrillation	134	31.6%
Ischemic heart disease	279	65.8%
Myocardial infarction	99	23.3%
**Lifestyle**, ***N*** **(%)**		
Obesity	149	35.1%
Smoker	109	25.7%
Heavy drinker	39	9.2%

** IQR, Interquartile range (Q1–Q3).

### Post-stroke early seizures and epilepsy

During the follow-up period, a total of 121 patients experienced at least one epileptic seizure. Among these, 97 seizures were classified as early-onset, occurring relatively soon after the stroke, while 30 were considered late-onset seizures. Among the 30 late seizures, two cases were considered provoked seizures, indicating that they were triggered by specific identifiable causes or events. Six patients developed both early-onset and late-onset seizures.

#### Early seizure

Early seizures (ES) were focal in 76 cases (17.9% of all strokes), and in 18 cases (4.2% of all strokes), seizures presented with generalized features, focal-to-bilateral tonic-clonic seizures, or unknown onset. Status epilepticus occurred in three patients (0.7% of all strokes), in all cases as early seizures (Supplementary Table 1). Notably, in 82 cases (84.5% of all ES), the first early seizure developed within 24 h after the stroke onset. Nine (9) patients with ES and four patients without ES received anti-seizure medication (ASM) within the 1st week after admission, and one of these patients developed post-stroke epilepsy (PSE).

#### Post-stroke epilepsy

Thirty patients developed late seizures. However, in two cases, seizures were considered as provoked due to patients' severe condition and electrolyte imbalance. These patients died in hospital on days 8 and 22 and were not included in the PSE analysis. Five patients with PSE had recurrent seizures during follow-up despite being prescribed anti-seizure medication (ASM). One patient was prescribed ASM only after the second occurrence of late seizure and was seizure-free for the last follow-up period. Twenty-two patients prescribed ASM after the first episode of late seizure did not report recurrent seizures within the follow-up period.

**Figure 2:**
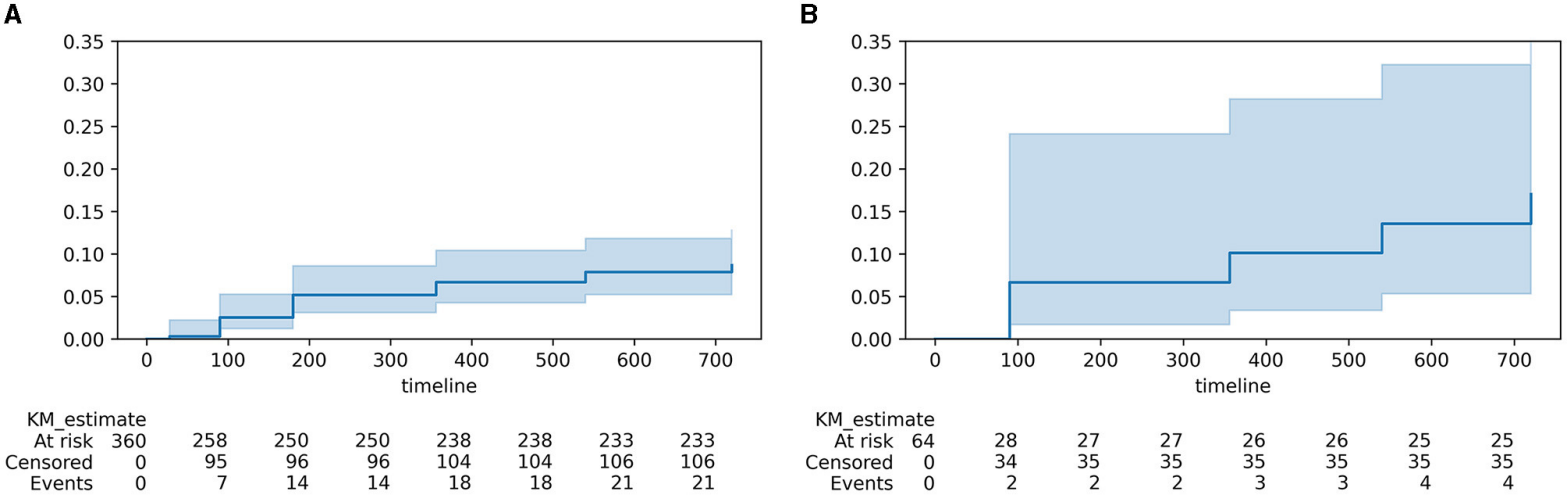
Kaplan–Meier curves for PSE in post-stroke patients. **(A)** ischemic strokes; **(B)** hemorrhagic strokes and infarctions with hemorrhagic component.

Using Kaplan–Meier regression, we calculated the 2-year cumulative risk of developing PSE. The overall 2-year cumulative risk for PSE was 9.7%. For ischemic strokes, the cumulative risk was 8.7%; for hemorrhagic strokes (intracranial parenchymal hemorrhage (ICH), subarachnoid hemorrhage (SAH), and secondary hemorrhagic IS combined), the 2-year cumulative risk was 15.4% (log-rank test, *p* = 0.01). Kaplan–Meier curves for PSE in these cohorts can be seen in Figure 2.

To identify risk factors for PSE after ischemic stroke, we analyzed a sample of 360 patients with ischemic strokes; ischemic strokes with hemorrhagic components were included with initial etiology. We did not analyze the risk factors of PSE in ICH and SAH since the number of cases was not sufficient. Significant differences were found between the PSE and non-PSE groups in terms of circulation territory involvement and characteristics of discharge state (as shown in Table 2). Interestingly, the occurrence of early seizures or seizures with convulsive symptoms was not associated with the development of PSE in the between-group analysis. Age also did not show a significant association with PSE development. A set of laboratory parameters was tested and analyzed within this study; the full list is provided in the Supplementary material. To identify a possible relationship between laboratory parameters and PSE, we applied Student's *t*-test and Mann–Whitney *U*-test for between-group analysis, and as a next step, Fine and Gray regression in a univariable mode. None of the laboratory parameters was significantly associated with PSE, and, therefore, we did not include them in the final multivariable Fine and Gray model. For more detailed information, see Supplementary Tables 2, 3.

**Table 2:** Differences between PSE and non-PSE groups for ischemic stroke patients.

**PSE in post-ischemic patients**	**Between-group analysis**				***p*-value**
	**Absent**, ***N*** = **337 (100%)**		**Present**, ***N*** = **23 (100%)**		
Age, mean (±SD)	67.6	±10.9	66.3	±11.7	0.40^†^
Male sex, *N* (%)	178	52.8%	11	47.8%	0.80^‡^
First-ever, *N* (%)	282	83.7%	20	87.0%	0.90^‡^
Secondary hemorrhagic, *N* (%)	12	3.6%	2	8.7%	0.50^‡^
Left lateralization, *N* (%)	177	52.5%	9	39.1%	0.30^‡^
Cortical involvement, *N* (%)	27	8.0%	1	4.3%	0.87^‡^
TOAST, N (% of all ischemic strokes)					0.98^‡^
Large artery	220	65.3%	14	60.9%	
Cardioembolism	70	20.8%	5	21.7%	
Lacunar	30	8.9%	3	13.0%	
Undetermined	14	4.2%	1	4.3%	
Circulation territory (for ischemic stroke), N (% of all ischemic strokes)					0.002^‡^
Anterior cerebral artery	19	5.6%	2	8.7%	0.88^‡^
Middle cerebral artery	301	89.3%	16	69.6%	0.01^‡^
Vertebral artery	8	2.4%	1	4.3%	0.92^‡^
Watershed	9	2.7%	4	17.4%	0.002^‡^
NIHSS at admission, median (IQR ^**^)	9	5.0–14.0	8	4.5–14.5	0.33^§^
Early-onset seizure (0–7 days), *N* (%)	71	21.1%	4	17.4%	0.88^‡^
Seizures with convulsive symptoms	22	6.5%	3	13.0%	0.20^‡^
Recurrent stroke, *N* (%)	32	9.5%	3	13.0%	0.85^‡^
**Vascular risk factors**, ***N*** **(%)**					
Diabetes mellitus	144	42.7%	7	30.4%	0.35^‡^
Dyslipidemia	159	47.2%	11	47.8%	0.88^‡^
Hypertension (160/100 and above)	303	89.9%	18	78.3%	0.16^‡^
Atrial fibrillation	115	34.1%	8	34.8%	0.87^‡^
Ischemic heart disease	230	68.2%	17	73.9%	0.74^‡^
Myocardial infarction	82	24.3%	6	26.1%	0.95^‡^
**Lifestyle**, ***N*** **(%)**					
Obesity	116	34.4%	11	47.8%	0.28^‡^
Smoker	88	26.1%	7	30.4%	0.83^‡^
Heavy drinker	30	8.9%	2	8.7%	0.73^‡^
**Status at discharge**					
Barthel Scale, median (IQR)	90	80.0–100.0	82, 5	52.5–95.0	0.08^§^
Rankin Scale, median (IQR)	2	1.0–3.0	2	1.0–3.0	0.28^§^
NIHSS, median (IQR)	2	1.0–6.0	4	2.00–9.75	0.11^§^

** IQR, Interquartile range (Q1–Q3).

† Student's t-test;

‡ Chi-square test with Yates's correction;

§ Mann–Whitney U-test.

Based on the between-group analysis, watershed stroke and lower Barthel index were identified as possible predictors of PSE and were selected for the final multivariable Fine and Gray analysis. Both parameters were revealed as independent predictors of developing PSE with Hazard Ratios 6.01 for watershed infarction (CI 2.29–15.77, *p* < 0.001) and 0.98 for Barthel index score at discharge (CI 0.96–0.99, *p* = 0.04).

**Figure 3:**
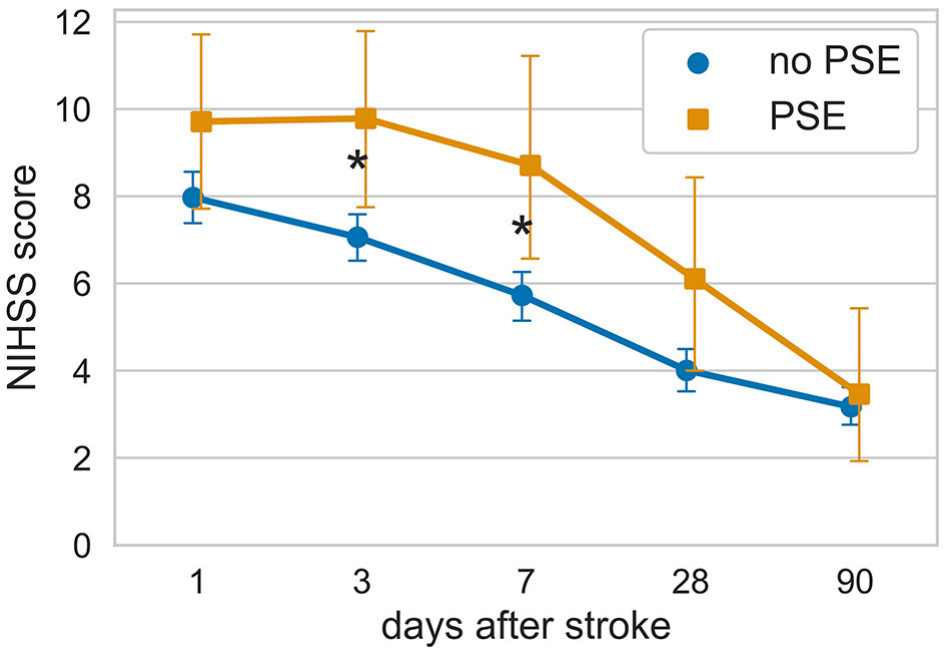
Evolution of neurological deficit in hospital survivors with and without subsequent PSE. *p < 0.05 according to the Mann–Whitney *U*-test after FDR correction for multiple comparisons (Benjamini/Hochberg method).

We collected repeated data on neurological recovery measured using the National Institute of Health Stroke Scale (NIHSS) during the hospitalization period. This allowed us to investigate the recovery dynamics for each patient. Interestingly, patients who later developed PSE exhibited a slower recovery time course during their inpatient stay, particularly during the first several days. The 1-month evolution of NIHSS scores differed significantly between stroke survivors with and without PSE (mixed ANOVA, for factor PSE *p*-value = 0.009), as shown in Figure 3.

### Survival

Out of the total cohort, 124 patients (30%) passed away during their hospital stay, and an additional 45 patients (11%) passed away in the 2-year follow-up period. Kaplan–Meier survival curves for the entire cohort, as well as for patients with ischemic and hemorrhagic strokes, are shown in Supplementary Figure 1. Patients with hemorrhagic stroke had a higher mortality rate in comparison to those with ischemic stroke (log-rank test, *p* < 0.001).

#### In-hospital mortality

In the between-group analysis, several factors were found to be associated with in-hospital mortality. These factors included older age (*p* < 0.001), female sex (*p* = 0.005), SAH stroke type (*p* < 0.001), cardioembolic stroke (*p* < 0.001), ES occurrence (*p* < 0.001), higher NIHSS at admission (*p* < 0.001), lower GCS score at admission (*p* < 0.001), atrial fibrillation (*p* < 0.001), a history of ischemic heart disease (*p* < 0.001), and a history of myocardial infarction (*p* = 0.045); data are shown in Supplementary Table 4. The multivariable Cox proportional hazard model identified older age (*p* = 0.01, HR 1.03, 95% CI 1.01–1.05), higher NIHSS score at admission (*p* < 0.005, HR 1.17, 95% CI 1.13–1.20), SAH (*p* = 0.001, HR 1.34, 95% CI 1.12–1.6), and ES (*p* = 0.05, HR 1.45, 95% CI 1.0–2.11) as independent risk factors for in-hospital mortality; data are shown in Supplementary Table 4 and Supplementary Figure 2.

#### Two-year mortality

In this study, the presence of post-stroke epilepsy (PSE) was not found to be associated with 2-year mortality: 39 patients (14%) died in the non-PSE cohort and 6 patients (21%) in the PSE cohort (*p* = 0.4 in Fisher's exact test). Between-group analyses for those with and without PSE for 2-year mortality did not reveal clinical predictors for long-term mortality, and data are shown in Supplementary Table 5.

## Discussion

In this prospective study conducted within a post-stroke cohort, the overall cumulative incidence of post-stroke epilepsy (PSE) within a 2-year post-stroke period was 9.7%. Specifically, after ischemic strokes, the incidence was 8.7%, and after hemorrhagic strokes, it was 15.4%. These estimates align with previous studies (5, 22–25). During the assessment of PSE incidence, we combined subarachnoid bleedings (SAH) and parenchymal hemorrhages (ICH). Although these stroke types may involve different processes in the formation of epileptic foci, they share more similarities with each other than with ischemic stroke in the context of epileptogenesis. Both SAH and ICH are considered significant risk factors for PSE (5, 22, 26–28), with hemorrhagic transformation of ischemic stroke also being an independent risk factor for PSE (29). Thus, hemorrhagic stroke has been consistently recognized as an independent risk factor for the development of late seizures as demonstrated through multivariable analysis with appropriate confounder adjustments.

We would like to focus on the reasons why we have decided to exclude 19 patients who underwent thrombolysis in the acute stroke period. As we wrote earlier, this approach to stroke treatment was not yet well established at our hospital during the study period. In addition, in this period of time (from 1 January 2013 to 31 December 2016), there was conflicting evidence on the risk of early and late post-stroke seizures in patients who underwent this procedure. De Reuck et al. (30) assumed that thrombolysis prevented partly the occurrence of late-onset seizures, while Iyer et al. (31) reported neurotoxic and epileptogenic properties of plasminogen activator. Modern studies demonstrate a lack of evidence of the influence of reperfusion therapies on the occurrence of early post-stroke seizures or the time to post-stroke epilepsy (32, 33).

Regarding identifying risk factors, we focused exclusively on patients with IS and did not include ICH and SAH cases. Our study identified watershed infarction and lower Barthel index at discharge as predictors of PSE development within 2 years after the ischemic stroke. A lower Barthel index at discharge has already been reported as an independent risk factor of PSE (7, 34). The Barthel index at discharge may indicate the amount of recovery for a patient during their hospitalization period. Previous studies have reported that individuals who subsequently developed PSE spent more days in the intensive care unit, potentially indicating a poorer post-stroke recovery (7, 35–38). Furthermore, in our study, within the PSE group, the National Institutes of Health Stroke Scale (NIHSS) scores did not show significant improvement during the first 7 days after the stroke, whereas neurological recovery in stroke survivors in the non-PSE group exhibited an early onset after the stroke. Thus, there appears to be an inverse relationship between the rate of improvement during the acute post-stroke period and the subsequent development of PSE.

Watershed stroke, a distinct subtype of ischemic stroke, is characterized by its occurrence in the border zones between two major cerebral arteries. In our study, we observed a substantial and robust relationship between watershed stroke and the development of PSE. Employing multivariable analysis, we identified watershed infarction as the most prominent and significant risk factor for PSE within our cohort. Two mechanisms are considered to be involved in watershed infarction: low-flow and micro-embolism (39). Clinical data indicate that outcomes of watershed strokes caused by these etiologies may differ in their clinical manifestations and prognosis (21). Watershed infarction could present as a stroke with slow progressive onset lasting from hours to days (40), potentially leading to delayed initiation of recovery. In our study, watershed stroke was found to be a strong and independent risk factor of PSE as demonstrated through multivariable Fine and Gray regression analysis. Indeed, the findings from several previous research studies are consistent with the results obtained in our study. Several studies have reported similar outcomes, supporting the association between watershed stroke and the development of PSE. MRI-confirmed stroke patients with watershed infarction were found to have a four-fold increased risk of early seizures compared to other cortical infarcts (41). Moreover, watershed strokes were more prevalent in patients experiencing both early and late post-stroke seizures compared to stroke patients without seizures (42). Furthermore, a retrospective study associated strokes classified under “other” etiologies according to the TOAST criteria, which may include watershed strokes, with an increased risk of PSE (43). Within our study, watershed stroke was significantly associated with PSE, demonstrating a relatively high hazard ratio of 6.01. Considering that the Fine and Gray model utilized in our analysis accounted for the competitive risk of death, this result suggests a substantial association between watershed stroke and PSE while also indicating a reduced mortality risk in patients with watershed infarction. It is crucial to highlight that the inclusion of mortality as a competing risk factor in the analysis while enhancing the comprehensiveness of the findings can also introduce a degree of variability in the results. In our study, employing a Cox proportional hazard model on a subset of patients who survived the first 7 post-stroke days revealed a hazard ratio (HR) of 2.2, with a 95% confidence interval of 1.6–3.1 and *p* < 0.005. This HR is notably lower than that obtained when applying the Fine and Gray model to the entire patient cohort. However, it is noteworthy that both modeling approaches, whether applied to the entire patient cohort or the subset of 7-day survivors, consistently point to the significant influence of watershed infarction in the development of post-stroke epilepsy. This demonstrates the robustness of our findings regarding the pivotal role of watershed infarction in this context. Thus, the collective body of evidence from previous studies, along with the findings of our study, highlights the considerable influence of watershed stroke as a critical risk factor for the development of PSE and underscores the importance of understanding the mechanisms and clinical implications of this stroke subtype in the context of post-stroke epilepsy.

In a comprehensive study involving 516 post-stroke patients, Pezzini et al. discerned the distribution of early ES wherein 41% were classified as focal, 27% as generalized, and 1% as status epilepticus (44). Conversely, our study's cohort exhibited a distinct pattern, with a heightened incidence of focal seizures and a diminished occurrence of seizures with generalized characteristics (ranging from focal to bilateral tonic-clonic or of unknown onset). Within our investigation, 63% of all early seizures were identified as focal aware, while 15.5% manifested as focal seizures without awareness; seizures with generalized features accounted for 18.5%, and status epilepticus was documented in 3% of all ES cases.

Within our study group, the incidence of individuals with ES constituted 23% of all cases and almost 17% among patients who survived the in-hospital period. Several investigations have similarly documented an incidence of 13–17% for ES or status epilepticus during the acute stroke phase (45–47) although most studies report a lower ES incidence. Our study's distinct findings can be attributed to the implementation of a more advanced methodology for seizure identification. In our prior unpublished study, the observed incidence of seizures in post-stroke patients was 4.6% for patients with IS and 9.0% for patients with ICH, which corresponds to other published data. However, as our investigation progressed, it became evident that with conventional practices, certain seizure occurrences might be overlooked. We therefore in the present study focused on the methodology of seizure identification. We firmly believe that our results closely approximate the genuine frequency of seizures following a stroke. Nevertheless, it is essential to acknowledge that the precise frequency can only be ascertained through continuous EEG monitoring extending over several days after the stroke (48). The additional training of medical personnel in seizure detection has improved the identification of predominantly mild focal seizures that might otherwise have gone unnoticed. High ES incidence could also be explained by a high proportion of severe strokes including ICH and SAH. The median NIHS score upon admission in the total cohort was 9, which is indicative of moderate-to-severe strokes. Seizures were systematically detected and proactively assessed by neurologists and nurses from the point of emergency admission to discharge. The ambulance teams were additionally interviewed to identify seizures before admission. In cases of uncertainty, a qualified epileptologist available 24/7 in Buyanov City Hospital was called to verify the seizure. Hence, the manifestation of ES was accurately and prospectively ascertained. This meticulous scrutiny conceivably contributed to the enhanced identification of individuals with focal seizures, particularly those with focal aware seizures. It stands to reason that such cases might have eluded detection in retrospective analyses, and even in prospective ones where early seizures are retroactively identified in which ES is identified retrospectively through a subsequent chart review since focal aware seizures could be overlooked. Thus, the documented ES frequency of 23% represents a true occurrence rate in circumstances where there exists a heightened vigilance toward their manifestation. Early seizures are frequently considered a risk factor for PSE (11, 19, 27, 49–51). However, in our examination, the presence of ES did not exhibit an association with the development of PSE. Our investigation aligns with several previous studies that failed to establish ES as an independent risk determinant for PSE (8, 36, 52–54). When accounting for all seizure events, encompassing not only generalized motor episodes, the occurrence of ES may be less important. An additional explanation could be the heightened mortality rate among patients afflicted with ES: within our study, 48% of all ES patients died shortly after the stroke onset. Population-based case–control studies have reported a two-fold elevation in mortality among patients with ES in comparison to controls matched for age, gender, and stroke severity (55–57).

As a part of this research, we investigated the determinants influencing both short-term and long-term mortality after a stroke. Notably, advanced age, occurrence of SAH, higher NIHSS at admission, and manifestation of ES have exhibited robust and statistically significant association with increased mortality during the in-hospital stay after adjustment for confounders: gender, cardioembolic stroke etiology, and ischemic heart disease in history. These findings highlight the significance of certain demographic, clinical, and stroke-specific characteristics in predicting in-hospital mortality. The identified risk factors can aid healthcare providers in risk stratification and may help inform treatment decisions to improve patient outcomes during hospitalization.

Early seizures that occur within hours and days after a stroke complicate the initial recovery phase and significantly increase the risk of death first weeks after the stroke. We estimated surveillances in the group of patients with Kaplan–Meyer regression and applied a log-rank test to compare groups with ES and without ES (data are shown in Supplementary Figure 2). At a 3-month time point, patients who experienced ES exhibited a survival rate of 49.5%, which, notably, was significantly lower than the 75% survival rate observed in patients who did not experience ES. Interestingly, while ES was associated with increased short-term mortality, their impact on long-term mortality appeared to be less consistent.

The multivariable Cox proportional hazard model provides a more comprehensive analysis, accounting for multiple factors simultaneously, which strengthens the validity and reliability of the results. Our results show that the presence of PSE does not have a significant impact on long-term mortality in this study cohort. While PSE can have considerable implications for the quality of life and wellbeing of affected individuals, it appears that it may not be a major contributing factor to overall mortality during the 2-year follow-up period in this specific population.

It is important to note that this study provides valuable insights into the risk factors of PSE and mortality, suggesting watershed infarction as a major contributor to the development of PSE after an ischemic stroke and ES occurrence emerges as a pivotal factor driving heightened short-term mortality. However, further research with larger sample sizes and longer follow-up periods is required to fully explore these relationships and reveal the potential underlying mechanisms.

### Advantages

This study has a prospective design with a comprehensive 2-year follow-up period, characterized by minimal loss to follow-up among post-stroke survivors. Clinical and laboratory parameters were assessed in this study. The involvement of trained staff and the 24/7 availability of epileptologists allowed more exhaustive and accurate identification of early-onset seizures.

### Limitations

We were able to collect NIHSS through in-hospital assessments and the 3-month visit for all surviving patients. However, not all patients could be physically present for all the scheduled time points due to their inability to travel to the hospital. For patients who could not make in-person visits at the 6, 12, 18, and 24-month time points, we conducted telephone interviews. Given that the accurate assessment of NIHSS requires an in-person examination, we made the decision to exclude NIHSS data obtained from the 6- to 24-month time points from the analysis to maintain the integrity of our results and prevent potential bias. The effect of ASM on the course of PSE, as well as seizure frequency, was beyond the scope of this study. Additionally, we did not evaluate the impact of medications such as statins, oral anticoagulants, or antiplatelet medications.

## Conclusion

In this prospective hospital-based study, the cumulative incidence for PSE was higher after HS than after IS. Watershed stroke and low Barthel index at discharge were independent predictors for PSE. In patients who subsequently developed PSE, the post-stroke neurological recovery was delayed as compared to patients without PSE. Additional training for medical staff and careful examination of medical records and ambulance teams at patients' admission allow us to detect early seizure frequency in 23% of all stroke patients. The frequency of early seizures is higher than reported in other studies, which indicates that the real frequency of early post-stroke seizures might be underestimated by clinical staff.

## Data availability statement

The original contributions presented in the study are included in the article/Supplementary material; further inquiries can be directed to the authors.

## Ethics statement

The studies involving humans were approved by the Medical Ethical Committee of Buyanov City Hospital. The studies were conducted in accordance with the local legislation and institutional requirements. The participants provided their written informed consent to participate in this study.

## Author contributions

SF: Data curation, Formal analysis, Investigation, Methodology, Project administration, Visualization, Writing – original draft, Writing – review & editing. WH: Conceptualization, Supervision, Writing – review & editing. FR: Investigation, Writing – review & editing. SY: Data curation, Investigation, Writing – review & editing. OS: Project administration, Resources, Writing – review & editing. EV: Data curation, Writing – review & editing. IK: Data curation, Writing – review & editing. AS: Writing – review & editing. NG: Conceptualization, Supervision, Writing – review & editing. AG: Conceptualization, Funding acquisition, Resources, Supervision, Writing – review & editing.
