# Supplementary material for: Post-stroke seizures, epilepsy, and mortality in a prospective hospital-based study
Source: Front Neurol. 2023 Dec 1;14:1273270. doi: 10.3389/fneur.2023.1273270 (PMC10722584; doi:10.3389/fneur.2023.1273270)
Supplement: Supplementary file 1 [file Data_Sheet_1.docx]

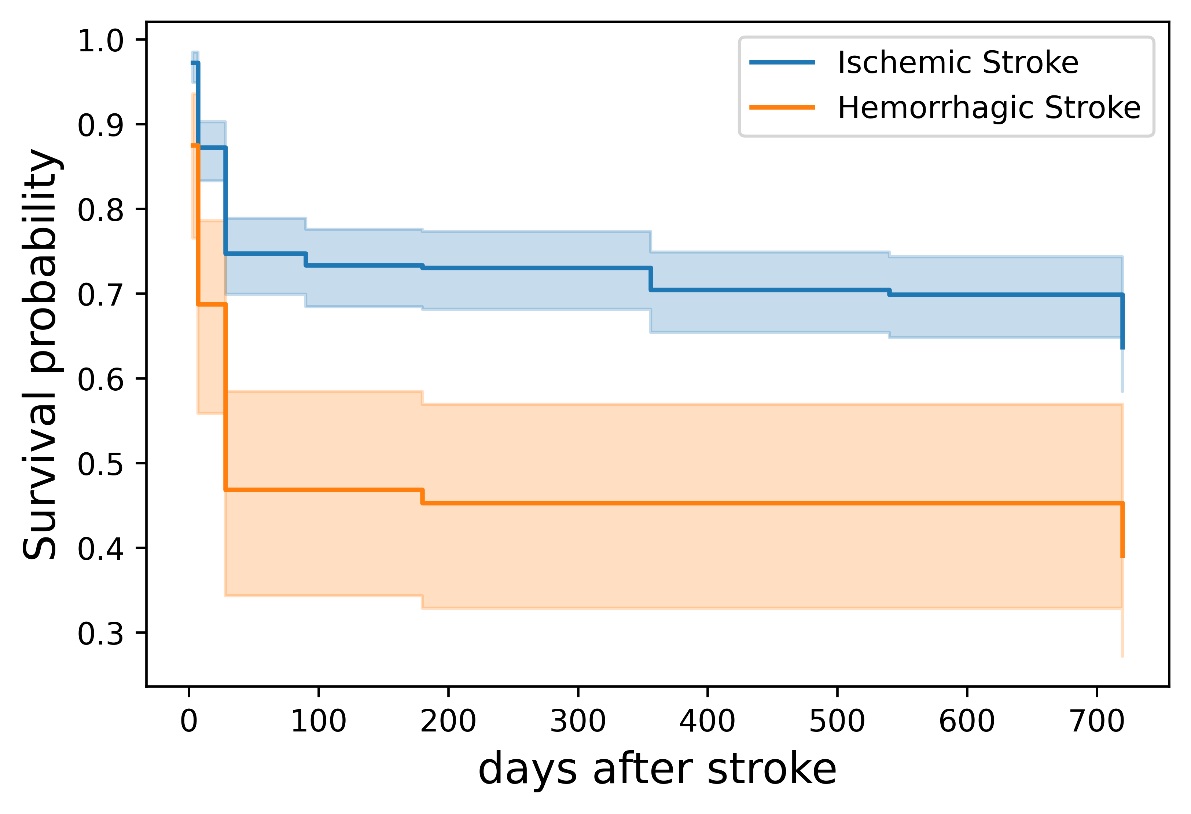


Supplementary Figure 1 Kaplan-Meyer curves for patients after ischemic strokes (IS) and hemorrhagic strokes (HS). IS includes IS and IS with secondary transformation; HS includes intracranial hemorrhages and subarachnoid hemorrhages.


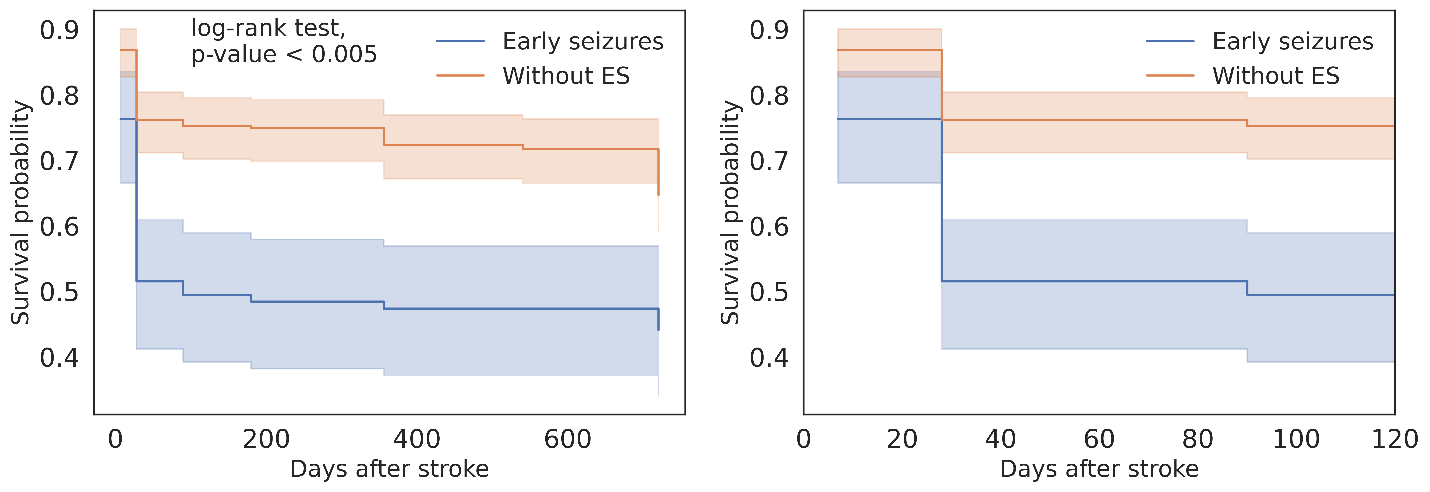


Supplementary Figure 2 Kaplan-Meyer curves for patients with and without early seizures (ES).

Supplementary Table 1. Early seizure types.

| **Early-onset seizure type, N (%)** | **Post-stroke patients (N=424)** | **In-hospital survivors (N=300)** | |
| --- | --- | --- | --- |
|  |  | Post-stroke epilepsy | |
|  |  | Absent, N=272 | Present, N=28 |
| Without early seizures | 327 (77.1%) | 226 (83.1%) | 24 (85.7%) |
| Focal aware | 61 (14.4%) | 33 (12.1%) | 2 (7.1%) |
| Focal with impaired awareness | 15 (3.5%) | 5 (1.8%) | 1 (3.6%) |
| Seizures with generalized features (focal to bilateral tonic-clonic seizures or unknown onset) | 18 (4.2%) | 7 (2.6%) | 1 (3.6%) |
| Status epilepticus | 3 (0.7%) | 1 (0.4%) | 0 |

| Supplementary Table 2 Differences between PSE and non PSE groups for ischemic stroke patients (N=360). | **Post-ischemic patients (N=360)** | | | | | | |
| --- | --- | --- | --- | --- | --- | --- | --- |
|  | total N | No late seizures | | Late seizures, mean (CI) | | Univariable, p-value | |
|  |  | mean | SD | mean | SD |  |  |
| Cl, mmol/L | 293 | 105,8 | 8,4 | 105,2 | 6,5 | 0,441 | § |
| K, mmol/L | 313 | 4,3 | 0,6 | 4,4 | 0,6 | 0,148 | § |
| Na, mmol/L | 312 | 141,7 | 4,9 | 140,8 | 3,5 | 0,374 | § |
| Hematocrit, % | 218 | 40,8 | 5,7 | 43,0 | 2,9 | 0,183 | † |
| Lymphocytes, % | 204 | 19,7 | 9,7 | 22,7 | 10,3 | 0,293 | † |
| Monocytes, % | 204 | 4,9 | 2,8 | 5,7 | 3,9 | 0,349 | § |
| Neutrophils, % | 167 | 67,6 | 11,7 | 62,5 | 12,2 | 0,184 | † |
| Platelet, 10^3/µL | 344 | 221,2 | 74,6 | 219,4 | 90,4 | 0,248 | § |
| RBC, 10^12/µL | 344 | 4,6 | 0,6 | 4,8 | 0,7 | 0,185 | § |
| ALT, U/L | 326 | 32,7 | 31,5 | 33,6 | 27,1 | 0,429 | § |
| AST, U/L | 292 | 37,1 | 50,6 | 34,1 | 31,9 | 0,242 | § |
| Bilirubin (total), mmol/L | 273 | 13,3 | 10,1 | 14,9 | 8,9 | 0,171 | § |
| Cholesterol, mmol/L | 245 | 5,5 | 1,3 | 5,0 | 1,0 | 0,137 | † |
| LDH (total), U/L | 273 | 466,4 | 232,6 | 464,3 | 179,7 | 0,482 | § |
| Triglycerides, mmol/L | 155 | 1,8 | 0,9 | 1,5 | 0,7 | 0,088 | § |
| Urea, mmol/L | 330 | 8,1 | 5,6 | 6,7 | 2,1 | 0,130 | § |
| VLDL cholesterol, mmol/L | 145 | 0,8 | 0,5 | 0,6 | 0,2 | 0,079 | § |
| LDL cholesterol, mmol/L | 138 | 3,2 | 1,2 | 3,0 | 0,8 | 0,509 | † |
| HDL cholesterol, mmol/L | 153 | 1,4 | 0,4 | 1,3 | 0,5 | 0,659 | † |
| Fibrinogen, g/L | 213 | 3,3 | 0,7 | 3,1 | 0,5 | 0,259 | † |
| Prothrombin time, % | 291 | 83,0 | 21,9 | 80,4 | 20,9 | 0,354 | § |
| Thrombin time, sec | 197 | 22,9 | 14,9 | 20,7 | 4,3 | 0,270 | § |
| Thromboplastin time, sec | 298 | 38,2 | 18,6 | 41,7 | 29,1 | 0,383 | § |
| Serum glucose, mmol/L | 304 | 7,3 | 3,0 | 7,0 | 3,0 | 0,284 | § |
| Serum total protein, g/L | 300 | 70,3 | 7,1 | 70,4 | 5,0 | 0,975 | † |
| **† - Student t-test; § - Mann-Whithey U-test** | | | | | | | |

¶ RBC - red blood cells; ALT - alanine aminotransferase; AST - aspartate aminotransferase; LDH - lactate dehydrogenase; VLDL - very-low-density lipoprotein; LDL - low-density lipoprotein; HDL - high-density lipoprotein.

Supplementary Table 3. Results of univariable Fine and Gray model for laboratory parameters.

| Parameter | p-value | Hazard Ratio | 95% CI for HR |
| --- | --- | --- | --- |
| Triglycerides | 0.27 | 0.58 | 0.22-1.53 |
| VLDL cholesterol | 0.11 | 0.16 | 0.02-1.47 |

Supplementary Table 4. Between-group analysis for in-hospital mortality.

|  | **Discharged a1ive, n=300** | **In hospital deaths, n=124** | **Univariate, p-value** | |
| --- | --- | --- | --- | --- |
| Age, mean (CI) | 65.1 (±10.8) | 70.8 (±10.7) | <0.001 | † |
| Male sex, N (%) | 171 (57%) | 52 (42%) | 0.005 | ‡ |
| Ischemic stroke | 255 (85%) | 88 (71%) | 0.002 | ‡ |
| Hemorrhagic stroke | 45 (15%) | 36 (29%) |  |  |
| Hemorrhagic stroke, N (% of all HS) | | | <0.001 | ‡ |
| - ISHT | 14 (31%) | 2 (6%) | 0.005 | ‡ |
| - ICH | 26 (58%) | 11 (31%) | 0.024 | ‡ |
| - SAH | 5 (11%) | 23 (64%) | <0.001 | ‡ |
| First-ever, N (%) | 248 (83%) | 100 (81%) | 0.677 | ‡ |
| Left lateralization, N (%) | 157 (52%) | 66 (53%) | 0.915 | ‡ |
| Cortical involvement, N (%) | 131 (44%) | 75 (60%) | 0.129 | ‡ |
| TOAST, N (% of all ischemic strokes) |  |  | <0.001 | ‡ |
| - large artery | 145 (56%) | 28 (32%) | <0.001 | ‡ |
| - cardioembolism | 59 (23%) | 39 (44%) | <0.001 | ‡ |
| - lacunar | 34 (13%) | 1 (1%) | <0.001 | ‡ |
| - undetermined | 31 (12%) | 22 (25%) | 0.006 | ‡ |
| Circulation territory (for ischemic strokes), N (% of all ischemic strokes) | | | 0.561 | ‡ |
| - anterior cerebral artery | 10 (4%) | 3 (3%) |  |  |
| - middle cerebral artery | 236 (93%) | 82 (93%) |  |  |
| - vertebral artery | 7 (3%) | 3 (3%) |  |  |
| - watershed | 16 (6%) | 2 (2%) |  |  |
| Early-onset seizure (0-7 days), N (%) | 50 (17%) | 47 (38%) | <0.001 | ‡ |
| NIHSS, median (IQR¶) | 7 (4-11) | 18 (13-22) | <0.001 | § |
| Glasgow Coma Scale, median (IQR) | 15 (15-15) | 13 (9.5-15) | <0.001 | § |
| Vascular risk factors | | | | |
| - diabetes mellitus, N (%) | 125 (42%) | 47 (38%) | 0.515 | ‡ |
| - dyslipidemia, N (%) | 149 (50%) | 50 (40%) | 0.087 | ‡ |
| - hypertension (160/100 and above), N (%) | 268 (89%) | 113 (91%) | 0.724 | ‡ |
| - atrial fibrillation, N (%) | 79 (26%) | 55 (44%) | <0.001 | ‡ |
| - ischemic heart disease, N (%) | 182 (61%) | 97 (78%) | <0.001 | ‡ |
| - myocardial infarction, N (%) | 62 (21%) | 37 (30%) | 0.045 | ‡ |
| Lifestyle | | | | |
| - obesity, N (%) | 106 (35%) | 43 (35%) | 0.912 | ‡ |
| - smoker, N (%) | 95 (32%) | 14 (11%) | <0.001 | ‡ |
| - heavy drinker, N (%) | 29 (10%) | 10 (8%) | 0.713 | ‡ |
| **†- Student's t-test; ‡ - Fisher’s exact test; § - Mann-Whitney U-test** | | | | |
| ¶**IQR - - Interquartile Range (Q1 - Q3)** | | | | |

Supplementary Table 5. Between-group analysis for 2-year mortality.

|  | **Alive at 2 years, n=255** | **Dead at 2 years, n=45** | **Univariate, p-value** | |
| --- | --- | --- | --- | --- |
| Age, mean (CI) | 65.1 (±10.6) | 64.8 (±11.8) | 0.824 | † |
| Male sex, N (%) | 144 (56%) | 27 (60) | 0.745 | ‡ |
| Ischemic stroke | 216 (85%) | 39 (87%) | 0.825 | ‡ |
| Hemorrhage stroke | 39 (15%) | 6 (13%) |  |  |
| First-ever, N (%) | 208 (82%) | 40 (89%) | 0.289 | ‡ |
| Left lateralization, N (%) | 137 (54%) | 20 (44%) | 0.260 | ‡ |
| Cortical involvement, N (%) | 116 (45%) | 15 (33%) | 0.314 | ‡ |
| TOAST, N (% of all ischemic strokes) |  |  | 0.541 | ‡ |
| - large artery, N (%) | 125 (58%) | 20 (51%) |  | |
| - cardioembolism, N (%) | 52 (24%) | 7 (18%) |  |  |
| - lacunar, N (%) | 27 (13%) | 7 (18%) |  |  |
| - undetermined, N (%) | 25 (12%) | 6 (15%) |  |  |
| Circulation territory (for ischemic strokes), N (% of all ischemic strokes) | | | 0.189 | ‡ |
| - anterior cerebral artery, N (%) | 8 (4%) | 2 (5%) |  | |
| - middle cerebral artery, N (%) | 204 (94%) | 32 (82%) |  |  |
| - vertebral artery, N (%) | 6 (3%) | 1 (3%) |  |  |
| - watershed, N (%) | 11 (5%) | 5 (13%) |  |  |
| Early-onset seizure (0-7 days), N (%) | 44 (17%) | 6 (13%) | 0.665 | ‡ |
| NIHSS, median (IQR¶) | 7 (4-11) | 7 (4-10) | 0.307 | § |
| Glasgow Coma Scale, median (IQR) | 15 (15-15) | 15 (15-15) | 0.430 | § |
| Vascular risk factors | | | | |
| - diabetes mellitus, N (%) | 108 (42%) | 17 (38%) | 0.625 | ‡ |
| - dyslipidemia, N (%) | 124 (49%) | 25 (56%) | 0.422 | ‡ |
| - hypertension (160/100 and above), N (%) | 225 (88%) | 43 (96%) | 0.192 | ‡ |
| - atrial fibrillation, N (%) | 72 (28%) | 7 (16%) | 0.096 | ‡ |
| - ischemic heart disease, N (%) | 154 (60%) | 28 (62%) | 0.870 | ‡ |
| - myocardial infarction, N (%) | 52 (20%) | 10 (22%) | 0.842 | ‡ |
| Lifestyle | | | | |
| - obesity, N (%) | 88 (35%) | 18 (40%) | 0.501 | ‡ |
| - smoker, N (%) | 77 (30%) | 18 (40%) | 0.224 | ‡ |
| - heavy drinker, N (%) | 23 (9%) | 6 (13%) | 0.409 | ‡ |
| Status at discharge |  |  |  |  |
| - Barthel Scale, median (IQR) | 90 (80-100) | 90 (70-100) | 0.385 | § |
| - Rankin Scale, median (IQR) | 2 (1-3) | 2 (1-3) | 0.419 | § |
| - NIHSS, median (IQR) | 2 (1-6) | 3 (1-6) | 0.365 | § |
| Post-stroke epilepsy, N(%) | 22 (9%) | 6 (13%) | 0.401 | ‡ |
| **Those who died in the hospital were not included in the assessment** | | | | |
| **†- Student's t-test; ‡ - Fisher’s exact test; § - Mann-Whitney U-test** | | | | |
| ¶**IQR - - Interquartile Range (Q1 - Q3)** | | | | |
